# *Schistosoma mansoni* Coinfection Attenuates Murine *Toxoplasma gondii*-Induced Crohn's-Like Ileitis by Preserving the Epithelial Barrier and Downregulating the Inflammatory Response

**DOI:** 10.3389/fimmu.2019.00442

**Published:** 2019-03-18

**Authors:** Beatriz Pêgo, Cesonia A. Martinusso, Claudio Bernardazzi, Beatriz Elias Ribeiro, Aline Fernandes de Araujo Cunha, Jacilene de Souza Mesquita, Hayandra F. Nanini, Marcelo Pelajo Machado, Morgana T. L. Castelo-Branco, Marta Guimarães Cavalcanti, Heitor S. P. de Souza

**Affiliations:** ^1^Internal Medicine, Federal University of Rio de Janeiro, Rio de Janeiro, Brazil; ^2^Institute of Microbiology Paulo de Góes, Federal University of Rio de Janeiro, Rio de Janeiro, Brazil; ^3^Laboratory of Microbiology and Parasitology, Federal University of Rio de Janeiro, Macaé, Brazil; ^4^Pathology Laboratory, IOC/FIOCRUZ, Rio de Janeiro, Brazil; ^5^Institute of Biomedical Sciences, Federal University of Rio de Janeiro, Rio de Janeiro, Brazil; ^6^Infectious Diseases Clinic, Federal University of Rio de Janeiro, Rio de Janeiro, Brazil; ^7^D'Or Institute for Research and Education (IDOR), Rio de Janeiro, Brazil

**Keywords:** *S. mansoni* coinfection, *T. gondii*-induced ileitis, mucosal immunity, Paneth cells, intestinal epithelial barrier, Crohn's disease

## Abstract

**Background and aims:** Mice orally infected with *T. gondii* develop Crohn's disease (CD)-like enteritis associated with severe mucosal damage and a systemic inflammatory response, resulting in high morbidity and mortality. Previously, helminthic infections have shown therapeutic potential in experimental colitis. However, the role of *S. mansoni* in *T. gondii*-induced CD-like enteritis has not been elucidated. Our study investigated the mechanisms underlying *T. gondii*-induced ileitis and the potential therapeutic effect of *S. mansoni* coinfection.

**Methods:** C57BL/6 mice were infected by subcutaneous injection of cercariae of the BH strain of *S. mansoni*, and 7–9 weeks later, they were orally infected with cysts of the ME49 strain of *T. gondii*. After euthanasia, the ileum was removed for histopathological analysis; staining for goblet cells; immunohistochemistry characterizing mononuclear cells, lysozyme expression, apoptotic cells, and intracellular pathway activation; and measuring gene expression levels by real-time PCR. Cytokine concentrations were measured in the serial serum samples and culture supernatants of the ileal explants, in addition to myeloperoxidase (MPO) activity.

**Results:** *T. gondii*-monoinfected mice presented dense inflammatory cell infiltrates and ulcerations in the terminal ileum, with abundant cell extrusion, apoptotic bodies, and necrosis; these effects were absent in *S. mansoni*-infected or coinfected animals. Coinfection preserved goblet cells and Paneth cells, remarkably depleted in *T. gondii*-infected mice. Densities of CD4- and CD11b-positive cells were increased in *T. gondii*- compared to *S. mansoni*-infected mice and controls. MPO was significantly increased among *T. gondii*-mice, while attenuated in coinfected animals. In *T. gondii*-infected mice, the culture supernatants of the explants showed increased concentrations of TNF-alpha, IFN-gamma, and IL-17, and the ileal tissue revealed increased expression of the mRNA transcripts for *IL-1 beta, NOS2, HMOX1, MMP3*, and *MMP9* and activation of NF-kappa B and p38 MAPK signaling, all of which were counterregulated by *S. mansoni* coinfection.

**Conclusion:** *S. mansoni* coinfection attenuates *T. gondii*-induced ileitis by preserving mucosal integrity and downregulating the local inflammatory response based on the activation of NF-kappa B and MAPK. The protective function of prior *S. mansoni* infection suggests the involvement of innate immune mechanisms and supports a conceptually new approach to the treatment of chronic inflammatory diseases, including CD.

## Introduction

Inflammatory bowel disease (IBD) constitutes a complex and heterogeneous disorder of the gastrointestinal tract, comprising both Crohn's disease (CD) and ulcerative colitis (UC). Despite the recent progress achieved in understanding IBD pathogenesis, encompassing genetic predisposition, and the interactions between the immune system and environmental and microbial elements, the cause of disease is still unclear, and therapy remains mostly empirical with limited effectiveness (1). Although the available data from developing countries are still limited, there is currently a general consensus that the incidence and prevalence of IBD are progressively increasing, and IBD is emerging as a worldwide disease (2). In fact, the results from a recent systematic review indicate an accelerating incidence in countries that are becoming more industrialized and more westernized, corroborating the notion of a global distribution of IBD (3).

Nevertheless, it is interesting to notice that the epidemiological evidence also unveils a link between the reduction in the burden of infectious diseases and the emergence of allergic and immune-mediated chronic inflammatory disorders (4). In this context, while the incidence of CD has been associated with improvements in socioeconomic status, fewer infections and high domestic hygiene in childhood (5), it has been inversely correlated with the distribution of helminthic infections (6, 7). These findings are compatible with the hygiene hypothesis, in which environmental elements, including the microbiota, are essential to the education of the immune system beginning very early in life (8). In the last few years, an evolutionary mismatch, particularly the loss of helminths from the human biome, has been proposed to be the underlying cause of the widespread increase in immune-related disorders (9). One mechanistic explanation of the potential protection conferred by helminth worm parasites from allergic and inflammatory conditions is that helminths can modulate the immune response, preventing the host from eliminating the parasites while concomitantly decreasing the reactivity against other pathogens (10). The shift in the immune reactivity caused by helminth infections is characterized by the silencing of the proinflammatory Th1 and Th17 T-cell subsets (11), and a synchronous promotion of regulatory T cells (Tregs) (12), including a prominent role for the regulatory cytokine interleukin (IL)-10 (13).

Attempts to treat CD and other autoimmune diseases using helminths to downregulate the inflammatory response, especially responses based on Th1 and Th17 immunity, have been reported occasionally (14). In human CD, pilot studies reported the beneficial effects of administering *Trichuris suis* ova to patients, indicating a possible downregulation of aberrant intestinal inflammation and reinforcing the notion that natural exposure to helminths may afford protection from immune-mediated diseases (15). Moreover, in another pilot study using helminths to treat CD, the investigators also found significant clinical improvement in patients infected with *Necator americanus* larvae (16). In experimental CD, using a model of 2,4,6-trinitrobenzene sulfonic acid (TNBS)-induced colitis, which prompts a predominant Th1-type immune response (17), investigators showed that helminthic infection with *S. mansoni* attenuates intestinal inflammation (18).

In the last three decades, animal models of intestinal inflammation have contributed important information on intestinal homeostasis and mucosal immunity, critical elements for the understanding of IBD pathogenesis. However, all models of IBD have limitations, and most of the models affect only the colon (17, 19). Previous studies have demonstrated that oral infection with *T. gondii* induces severe inflammation in the small intestine, and *T. gondii* infection has been proposed as a model for CD ileitis (20). The inflammatory response resulting from *T. gondii* infection involves the systemic and local induction of the Th1 response and of IL-23/IL-22 cytokines, which are implicated in the disruption of intestinal homeostasis, with an overexpression of matrix metalloproteinases (MMPs) and increasing host morbidity and mortality regardless of parasite control (21, 22). Considering that oral *T. gondii* infection fosters compartmental and systemic inflammatory responses resulting in intestinal tissue damage in mice resembling human CD ileitis, we hypothesized that prior infection with *S. mansoni* would render mice less susceptible to the development of enteritis.

## Materials and Methods

### Ethics Statement for Animal Studies

The institutional animal care committee of the Health Sciences Centre of the Federal University of Rio de Janeiro approved the care and use of the animals and procedures reported in this study (approval ID: 01200.001568/2013-87), in accordance with the guidelines of the International Care and Use Committee of the National Institutes of Health and the Guide for the Care and Use of Laboratory Animals (23).

### Animals

We utilized 6- to 8-week-old C57BL/6 mice, which were kept at constant temperature (25°C) in a room with a 12-h light-dark cycle. Standard laboratory pellet formula and tap water were provided *ad libitum*.

### Parasites and Infections

The *T. gondii* strain ME49 was kindly provided by Dr. R. Gazzinelli (UFMG, Minas Gerais, Brazil) and Dr. J. Lannes-Vieira (Fiocruz, Rio de Janeiro, Brazil). For oral infection, mice were infected by gavage using 100 ME49 cysts/animal. The cysts were obtained from C57BL/6 brain homogenates, as previously described (24). The *S. mansoni* strain BH was kindly provided by the Malacology Laboratory, Fiocruz (Rio de Janeiro, RJ, Brazil). To induce infection, 50 cercariae were inoculated in the back of each animal by subcutaneous injection.

### Experimental Design

After an acclimation period of 1 week, the mice maintained under specific pathogen-free conditions were randomly assigned to one of four groups, each containing 15 animals. Next, the animals of both the *S. mansoni* monoinfection and the coinfection groups were inoculated with *S. mansoni* cercariae. After 7 weeks, enteritis was induced through the introduction of *T. gondii* cysts by gavage in the animals of both the *T. gondii* monoinfection and the coinfection groups. A group of uninfected animals constituted the control group. Blood samples were collected from the tail vein at days 0, 4, and 7 after *T. gondii* infection. After harvesting, the blood samples were frozen and stored at −80°C for further analysis. For the surgical procedure, the animals were anesthetized subcutaneously with ketamine (35 mg/kg) and xylazine (5 mg/kg) and underwent a laparotomy performed with sterile technique. The ileal samples were opened longitudinally and rinsed in saline several times before being processed for the histological assessment and other studies. Histopathological analysis was also conducted on livers, due to the expected changes associated with the experimental model. The animals were euthanized by asphyxiation using increasing concentrations of CO_2_, and death was confirmed by cervical dislocation in experimental week 8 (seven days after *T. gondii* infection).

### Histological Assessment

The specimens were fixed in 40 g/L formaldehyde saline, embedded in paraffin, cut into 5-mm sections, stained with hematoxylin and eosin, and examined microscopically. The histological scores of the ileum were determined by two independent observers, as previously described (25). To further analyze the histopathological changes, we used the periodic acid-Schiff (PAS) technique to stain goblet cells within the intestinal epithelium. The density of the goblet cells was defined as the percentage of PAS-positive cells within at least 500 epithelial cells in the crypts and the surface epithelium of longitudinally sectioned intestinal pits. To assess eosinophils within the intestinal mucosa, sections were stained with Sirius red and counterstained with hematoxylin. Liver sections were stained with hematoxylin and eosin, and examined microscopically.

### Immunohistochemistry

Briefly, paraffin sections were cut onto slides pretreated with polylysine to characterize Paneth cells and intracellular signaling pathways using the indirect immunoperoxidase technique. Briefly, deparaffinized sections were first incubated at 90°C in 0.01 M sodium citrate buffer (pH 6.0) for 30 min for antigen retrieval. Then, the slides were immersed in 3% hydrogen peroxide in methanol for 10 min to block endogenous peroxidase activity. After being rinsed in phosphate-buffered saline (PBS) containing 0.5 % Tween 20 for 10 min, the tissue sections were incubated with non-immune horse serum for 30 min and subsequently, with the appropriate monoclonal antibody. Immunohistochemical staining was performed using the following primary antibodies: rabbit monoclonal anti-CD4 antibody (1:50; Santa Cruz Biotechnology Inc., Santa Cruz, CA); rabbit monoclonal anti-CD11b antibody (1:100; ab133357, Abcam, Cambridge, United Kingdom); rabbit polyclonal anti-lysozyme antibody (1:500; OriGene Technologies, Inc. Rockville, MD, USA); rabbit polyclonal anti-p38 [Thr180/Tyr182] (1:200) and anti-NFkB1/NFkB p105 (1:200) antibodies (both from Novus Biologicals, Littleton, CO, USA). Two sections from each sample were incubated with either PBS alone or with an isotype monoclonal IgG (concentration matched) and served as the negative controls. After incubation in a humidified chamber overnight at 4°C, the tissue sections were rinsed with PBS and incubated with a Dual Link System-HRP (Dako, Glostrup, Denmark) for 30 min at room temperature. Additional rinsing was followed by development with a solution containing hydrogen peroxide and diaminobenzidine (Dako, Glostrup, Denmark). The preparations were lightly counterstained in Harris's hematoxylin, dehydrated, and mounted in Permount (Fisher Scientific, Pittsburgh, PA, USA).

### Assessment of Apoptosis in the Ileum

Apoptosis was assessed in tissue sections of the ileum by a terminal deoxynucleotidyl transferase (TdT)-mediated dUTP nick-end labeling (TUNEL) assay. Samples from all experimental groups were analyzed using an ApopTag Peroxidase *in situ* Apoptosis Detection Kit (Millipore Corporation, Billerica, MA, USA). First, the paraffin sections were deparaffinized, hydrated, and incubated with a proteinase K solution. After blocking endogenous peroxidase activity, the slides were covered with the equilibration buffer and then incubated with a solution containing TdT enzyme. For the negative controls, we incubated a second section from each sample without TdT enzyme. For the positive controls, we pretreated samples with DNAse I (Sigma-Aldrich, Deisenhofen, Germany). After the reaction was terminated, the specimens were incubated with non-immune horse serum and then incubated with an anti-digoxigenin antibody peroxidase conjugate. As described above, the sections were developed with diaminobenzidine, counterstained in Harris's hematoxylin, dehydrated, and mounted in mounting medium. Morphologically preserved TUNEL-positive cells and apoptotic bodies were defined as apoptotic cells.

### Quantitative Assessment of Ileal Sections

The tissue sections were observed under a light microscope, and the quantitative analysis was carried out using a computer-assisted image analyzer (Leica QWin Plus V 3.5.1, Leica Microsystems Ltd, Switzerland). In the immunoperoxidase studies, the results of the quantitative analysis of the cell subsets were expressed as cell numbers per crypt (Paneth cells) or per mm^2^ lamina propria (eosinophils, CD4-, CD11b-, NF-kappa B-, and phospho-p38-positive cells). The density of apoptotic cells was defined by the number of immunoreactive cells in relation to total cells (immunoreactive and non-immunoreactive cells) in at least 500 epithelial cells in the crypts and the surface epithelium of longitudinally sectioned intestinal crypts. Two independent observers who were unaware of the experimental data examined all tissue sections and captured images.

### RNA Isolation, cDNA Synthesis, and Quantitative Real-Time PCR

The expression levels of selected genes were validated by quantitative real-time PCR (qRT-PCR). First, total RNA isolation from ileal specimens was performed using SV Total RNA isolation systems (Promega, Madison, WI, USA) following the manufacturer's protocol. A Nanodrop 2000 UV–Vis Spectrophotometer (Thermo Fisher Scientific, Wilmington, DE, USA) was used for quantifying and determining the purity of the RNA samples. Equal amounts of total RNA were reverse transcribed using a High-Capacity cDNA Archive kit. To quantify the mRNA, real-time RT-PCR was performed with an ABI Prism 7500 (Applied Biosystems, Foster City, CA, USA) using a CustomTaqMan® Array Gene Signature Plate (Thermo Scientific, Wilmington, DE, USA), including the *IL-4, IL-17A, IFN-gamma, IL-1 beta, IL- 22, TGF beta-1, MMP3, MMP9, HMOX1, NOS2, CCR5, CC3CR1, NF-kappa B*, and *MAPK14* genes. The mRNA levels were normalized to the expression levels of the control genes 18S and GAPDH. For the data analysis, the ΔΔCt method was used to determine the fold change of all of the target genes in each sample with 95 % confidence. The qRT-PCR reaction for each gene was performed in duplicate, and each experiment was repeated at least three times. The PCR cycles were performed according to the manufacturer's instructions.

### Organ Culture, Cytokine Measurements, and Aminotransferase Assays

Ileal explants were cultured in RPMI 1640 medium supplemented with 10 % fetal calf serum (Gibco-Invitrogen, Carlsbad, CA, USA), 2 mM L-glutamine, 50 mM 2-mercaptoethanol, 10 mM HEPES, penicillin (10,000 units/mL), and streptomycin (10 mg/mL) (all from Sigma Chemical Co., St. Louis, MO, USA) for 24 h at 37°C in a 5% CO_2_ humidified incubator. The samples were centrifuged, and the supernatants were aliquoted and stored at −80°C. The plasma samples obtained at different time points and explant culture supernatants were then subjected to cytokine measurement with a Cytometric Bead Array Mouse Th1/Th2/Th17 Cytokine Kit (BD Biosciences, San Jose, CA, USA), using a FACSCalibur flow cytometer (BD Biosciences, San Jose, CA, USA). The results were provided and analyzed with BD CBA Analysis software. The total protein content of the biopsy specimens was estimated using a Pierce BCA protein assay kit (Thermo Fisher Scientific, Rockford, IL, USA) and was used to normalize the results. To measure the liver-associated enzymes aspartate (AST) and alanine ALT) transaminase in serum (day 7), a protocol modified from commercial colorimetric kits (Sigma Chemical Co., St. Louis, MO, USA) was employed. Briefly, 20 μl of serum was added to each well containing 100 μl of a master reaction mix, including enzyme mix, developer, substrate, and assay buffer. Plates were protected from light, mixed and incubated at 37°C. After 5 min, the initial measurements were obtained, measuring the absorbance at 450 nm, for AST, and at 570 nm, for ALT. Measurements were taken every 5 min, until the value of the most active sample was greater than the value of the highest standard (10 nmole/well).

### Myeloperoxidase Activity Assessment

After being used in organ cultures, ileal samples were collected and frozen at −80°C until the extraction of myeloperoxidase (MPO) when they were homogenized in potassium phosphate buffer (pH 6.0), frozen and defrosted twice, homogenized again in the potassium phosphate buffer (pH 6.0) containing 0.5% hexadecyltrimethyl-ammonium bromide (Sigma Chemical Co., St. Louis, MO, USA), and centrifuged at 40,000 g for 30 min at 4°C. The supernatants were discarded and the insoluble pellets were rehomogenized in the potassium phosphate buffer (pH 6.0) containing 0.5% hexadecyltrimethyl-ammonium bromide. Ten microliters of the supernatants was added to a 96-well plate containing 290 μl of 50 mM potassic PBS (pH 6.0), 3 μl of substrate solution, containing 20 mg/ml o-dianisidine (Sigma Chemical Co., St. Louis, MO, USA), and 3 μl of H_2_O_2_ (20 mM). The plate components were rapidly mixed and the absorbance was determined at 460 nm for 1 min with a spectrophotometer. MPO activity was measured by a standard curve of the samples in units of MPO/mg of colonic samples.

### Statistical Analysis

Statistical analyses were performed using SPSS 20.0 software (SPSS Inc., Chicago, IL, USA). Statistical differences between the experimental groups were evaluated with the Mann–Whitney test or the Kruskal–Wallis ANOVA on ranks test, in which multiple comparisons were carried out using Dunnett's test, as appropriate. The values are expressed as the medians with interquartile ranges. The survival data are presented as a Kaplan-Meier survival curve and were analyzed with the log-rank test. For the serum cytokines, an analysis was performed by linear regression. All tests were two-tailed, and statistical significance was established as *P*-values < 0.05.

### Availability of Materials and Data

Materials, such as protocols, analytic methods, and study material, are available upon request to interested researchers. The raw data supporting the conclusions of this manuscript will be made available by the authors, without undue reservation, to any qualified researcher.

## Results

### *T. gondii* Monoinfection and Coinfection With *S. mansoni* Lead to Increased Morbidity and Mortality

**Figure 1:**
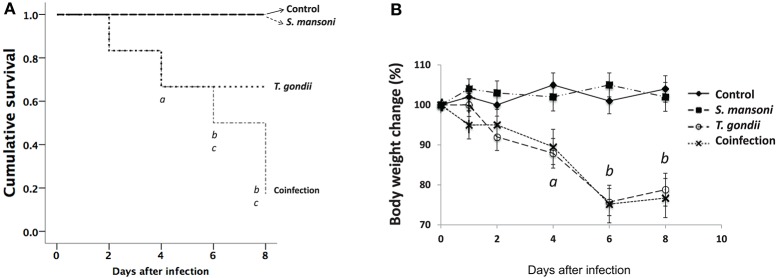
Increased morbidity and mortality in *T. gondii-*infected mice and in mice coinfected with *T. gondii* and *S. mansoni*. The survival curves for C57BL/6 mice **(A)** showed a significant reduction in the survival of mice infected with *T. gondii* alone compared with that of the controls and *S. mansoni*-infected animals (a, *p* < 0.001) and in the survival of mice coinfected with *T. gondii* and *S. mansoni* compared to that of the controls (b, *p* < 0.001) and *S. mansoni*-infected animals (c, *p* < 0.001). Morbidity was evaluated by determining the relative weight loss **(B)**. Mice infected with *T. gondii* alone and mice coinfected with *T. gondii* and *S. mansoni* presented progressive weight loss (a, *p* = 0.001; b, *p* < 0.001) compared with the controls and *S. mansoni*-infected mice. The data are representative of three independent experiments, with 5–7 animals per group. The survival curves were analyzed, and the *p*-values were determined by the log-rank test.

Following the same protocol of oral *T. gondii* infection, we have previously shown that C57BL/6 wild-type infected mice have increased mortality in association with the inflammatory response within the intestinal mucosa (26). In this study, we detected body weight loss as early as day two after *T. gondii* infection and increased mortality throughout the follow-up period. While monoinfection with *S. mansoni* did not affect the general health status of the animals, coinfected mice had weight loss similar to that of *T. gondii*-infected animals and a higher mortality rate than *S. mansoni*-monoinfected mice (Figure 1). The histopathological examination of liver sections from *T. gondii*-infected mice revealed the presence of foci of inflammation, with a relative preservation of the general tissue structure. *S. mansoni* monoinfection resulted in large multifocal eosinophilic granulomas, with the preservation of the essentially normal liver architecture. However, in coinfected mice, larger and less eosinophilic granulomas, surrounded by extensive areas of coagulative necrosis, and hepatocyte vacuolization were consistently observed. In parallel, significantly increased levels of AST and ALT, liver-associated enzymes, were detected in the serum of coinfected mice, compared to the other experimental groups. Levels in coinfected animals were almost twice as much of those observed among *T. gondii*-infected mice, measured at day 7 (Supplementary Figure S1). Together, histopathological findings and biochemical evidence for liver dysfunction support the notion of a synergistic effect of concurrent *S. mansoni* and *T. gondii* infections promoting severe liver damage. These results regarding hepatic damage may explain the higher morbidity and mortality observed among the coinfected animals.

### Prior Infection With *S. mansoni* Attenuates the Intestinal Injury From *T. gondii* Infection

**Figure 2:**
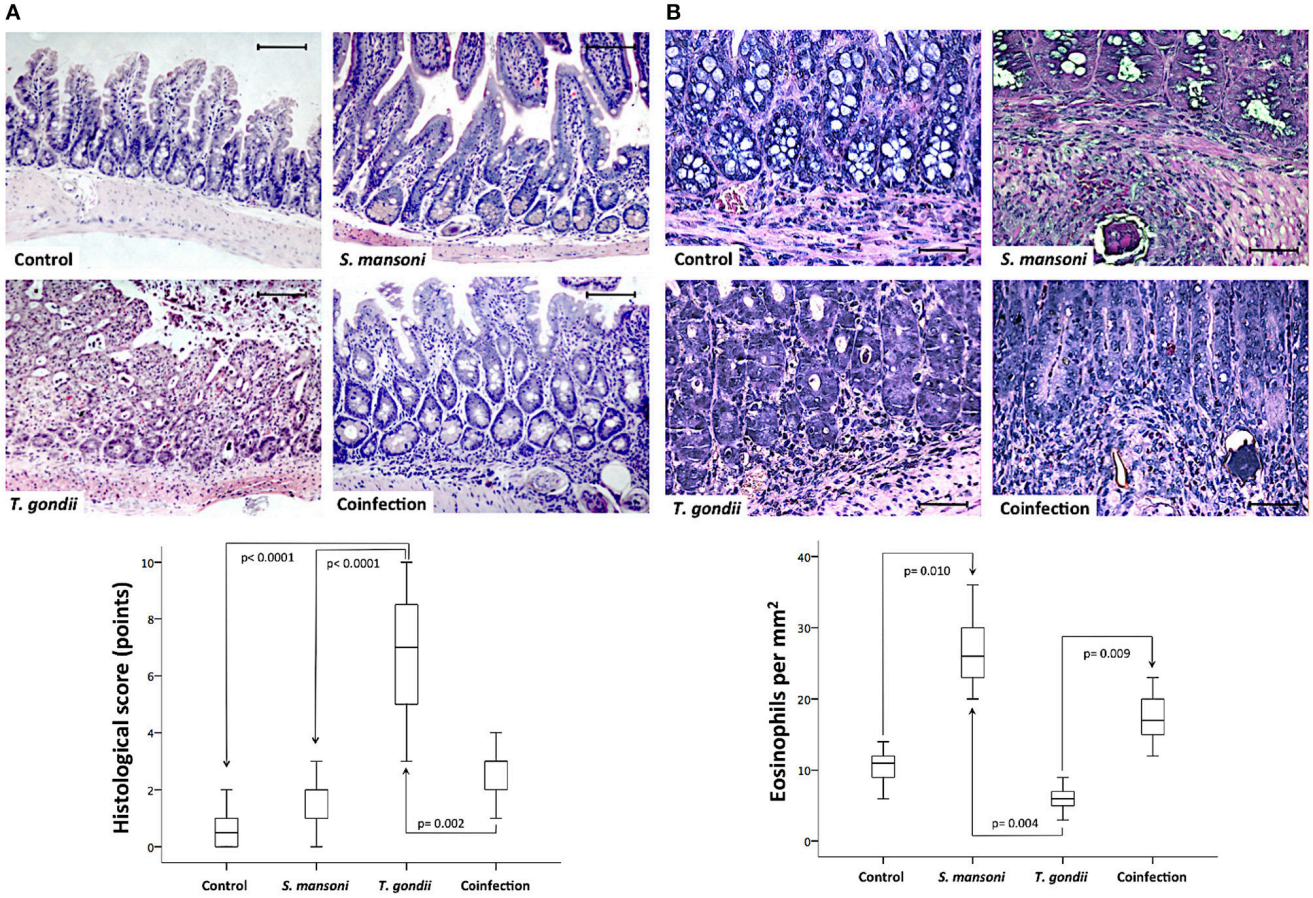
Prior *S. mansoni* infection attenuates *T. gondii*-induced inflammation and intestinal damage. Histopathological analysis by hematoxylin and eosin (HE) staining of the terminal ileum shows that *S. mansoni* infection prevents the severe inflammatory changes and tissue damage caused by *T. gondii* infection **(A)**. Sirius red staining revealed higher densities of eosinophils in *S. mansoni*-monoinfected or -coinfected animals compared with those from *T. gondii*-infected mice or non-infected controls **(B)**. The horizontal bars represent the medians, the boxes represent the 25th and 75th percentiles, and the vertical lines below and above the boxes represent the minimum and maximum values, respectively. The analysis was performed by Kruskal-Wallis ANOVA on ranks test, in which multiple comparisons were carried out using the Dunnett's test. The scale bars represent 20 μm. The data are representative of three independent experiments, with 5–7 animals per group.

Next, we investigated the effects of *T. gondii* and *S. mansoni* monoinfections and coinfection on intestinal injury. No histological damage was detected in the ileum of control and *S. mansoni*-monoinfected mice. *T. gondii*-induced ileitis manifested predominantly as continuous inflammatory lesions in the terminal ileum, including edema, ulceration, and evidence of transmural inflammation. Ileal samples from animals with concurrent infections with *S. mansoni* and *T. gondii* showed less inflammation compared to samples from *T. gondii*-monoinfected animals. Typically, intestinal damage in *T. gondii*-infected samples displayed several histological changes, including inflammatory infiltration of the entire lamina propria, blunting of the villi, abnormal crypt architecture, and areas of epithelial disruption, necrosis, and cell extrusion into the lumen. The tissue damage was determined by histopathological scores of the terminal ileum, and most samples from *T. gondii*-monoinfected mice presented significantly higher scores compared to samples from controls and *S. mansoni*-mono- and -coinfected animals (Figure 2A). To further characterize the nature of the inflammatory cell infiltrate within the intestinal lamina propria, we assessed eosinophils by immunohistochemistry. Ileal samples from *S. mansoni*-monoinfected or -coinfected animals showed significantly higher densities of eosinophils compared with those from *T. gondii*-infected mice or non-infected controls (Figure 2B).

**Figure 3:**
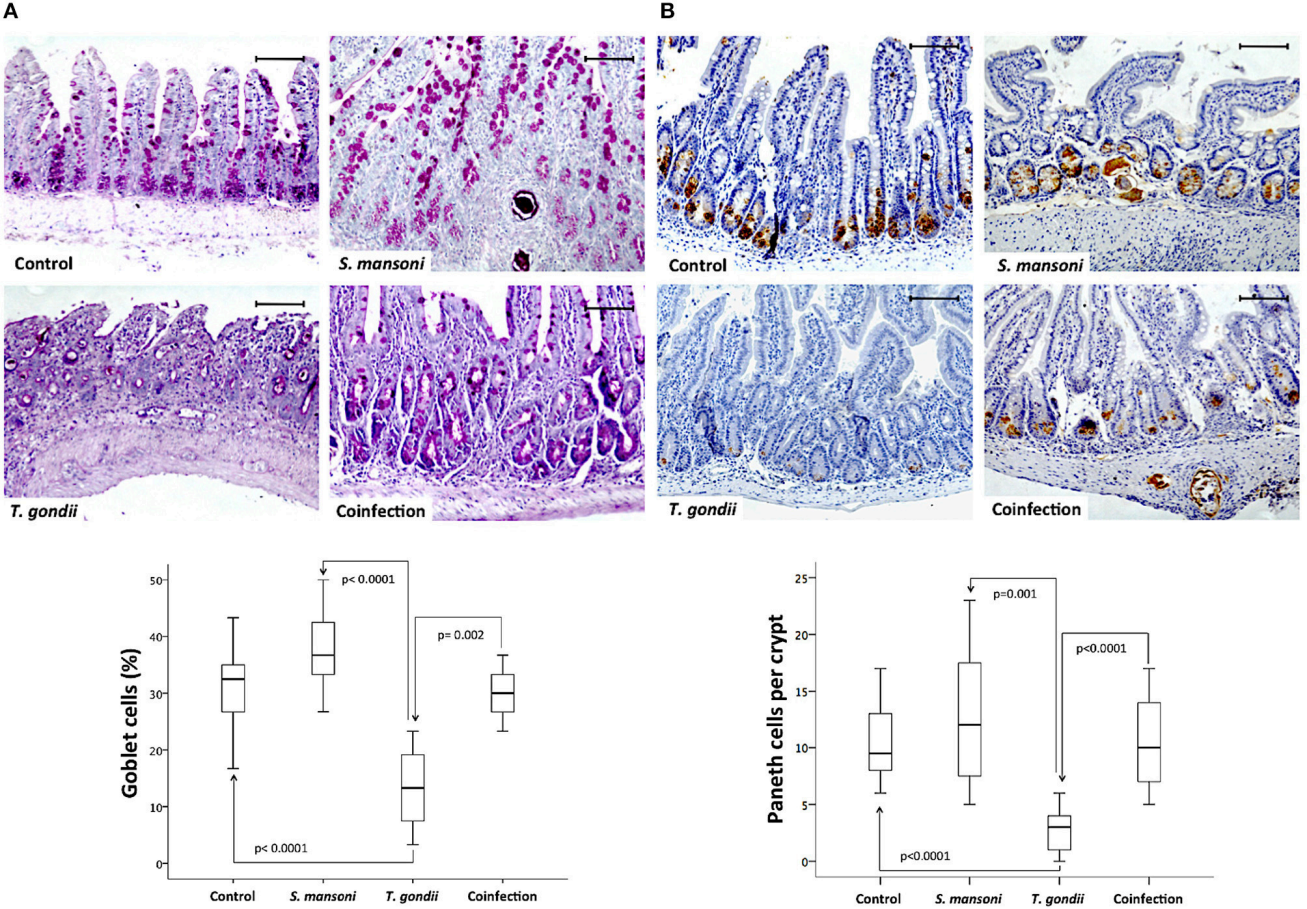
Prior *S. mansoni* infection preserves specialized epithelial cells in the ileum upon *T. gondii* challenge. Coinfection reduces the loss of goblet cells induced by *T. gondii* infection **(A)**. Coinfection also reduces the loss of Paneth cells induced by *T. gondii* infection **(B)**. The horizontal bars represent the medians, the boxes represent the 25th and 75th percentiles, and the vertical lines below and above the boxes represent the minimum and maximum values, respectively. The analysis was performed by Kruskal-Wallis ANOVA on ranks test, in which multiple comparisons were carried out using the Dunnett's test. The scale bars represent 20 μm. The data are representative of three independent experiments, with 5–7 animals per group.

In the epithelial compartment of the ileum, the density of mucous-secreting goblet cells was significantly lower in *T. gondii*-infected samples compared to samples from the normal control group or *S. mansoni*-monoinfected or -coinfected animals (Figure 3A). In addition, ileal samples from *T. gondii*-infected mice displayed significantly lower numbers of Paneth cells, labeled by immunohistochemistry with an anti-lysozyme antibody, compared to those from non-infected normal controls and *S. mansoni*-infected animals (Figure 3B).

**Figure 4:**
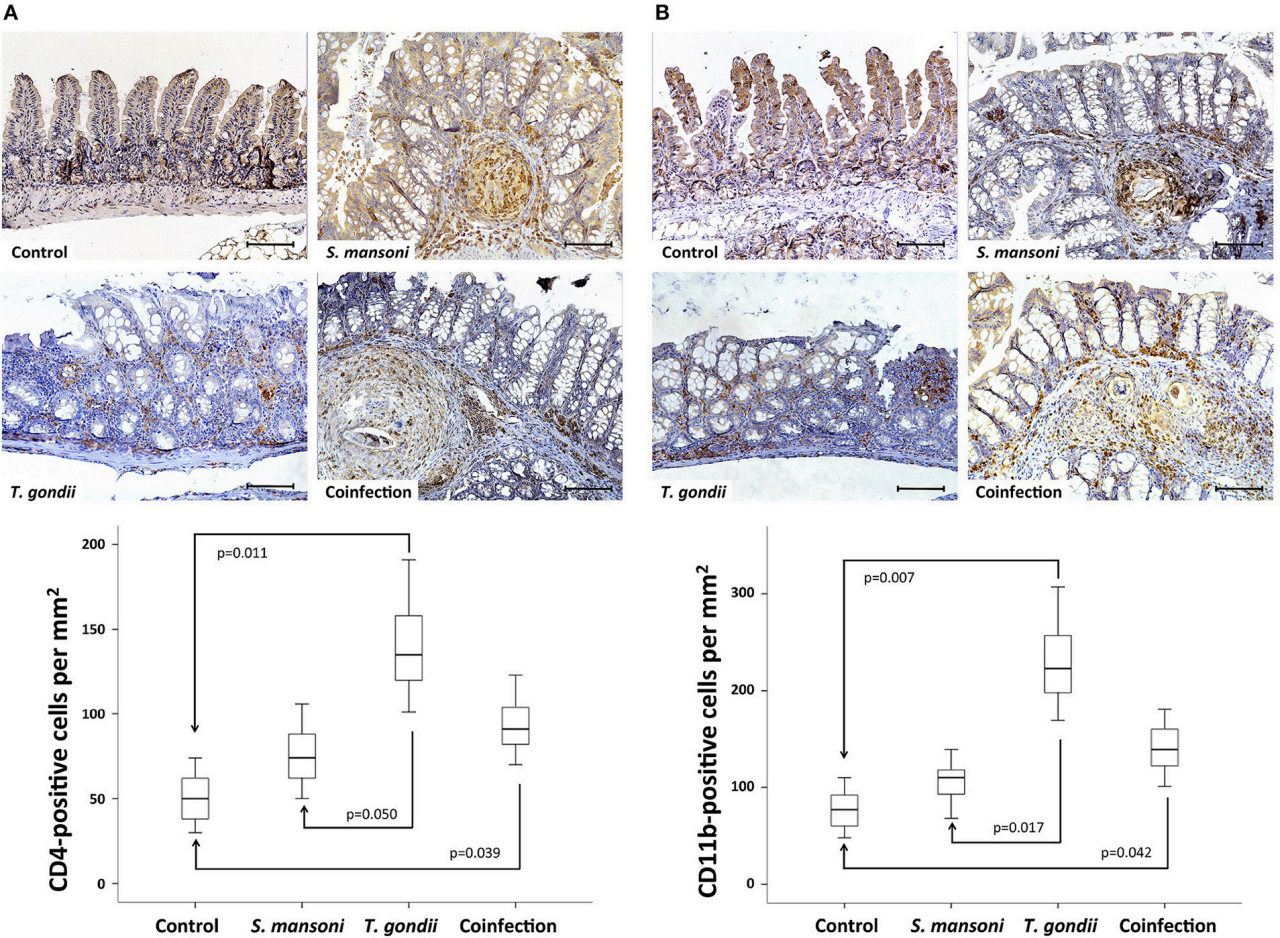
*T. gondii*-induced ileitis is characterized by an intense inflammatory cell infiltration in the lamina propria, including CD4- and CD11b-positive cells. Prior *S. mansoni* infection attenuates *T. gondii*-induced accumulation of CD4- **(A)** and CD11b-positive cells **(B)**. The horizontal bars represent the medians, the boxes represent the 25th and 75th percentiles, and the vertical lines below and above the boxes represent the minimum and maximum values, respectively. The analysis was performed by Kruskal-Wallis ANOVA on ranks test, in which multiple comparisons were carried out using the Dunnett's test. The scale bars represent 20 μm. The data are representative of three independent experiments, with 5–7 animals per group.

To characterize the different cell populations present in the lamina propria, we performed immunohistochemistry experiments to label CD4- and CD11b-positive cells. The dense inflammatory cell infiltration observed in the lamina propria of *T. gondii*-infected mice, comprised an increased concentration of both CD4- and CD11b-positive cells. Prior *S. mansoni* infection attenuates *T. gondii*-induced accumulation of CD4- (Figure 4A) and CD11b-positive cells (Figure 4B).

**Figure 5:**
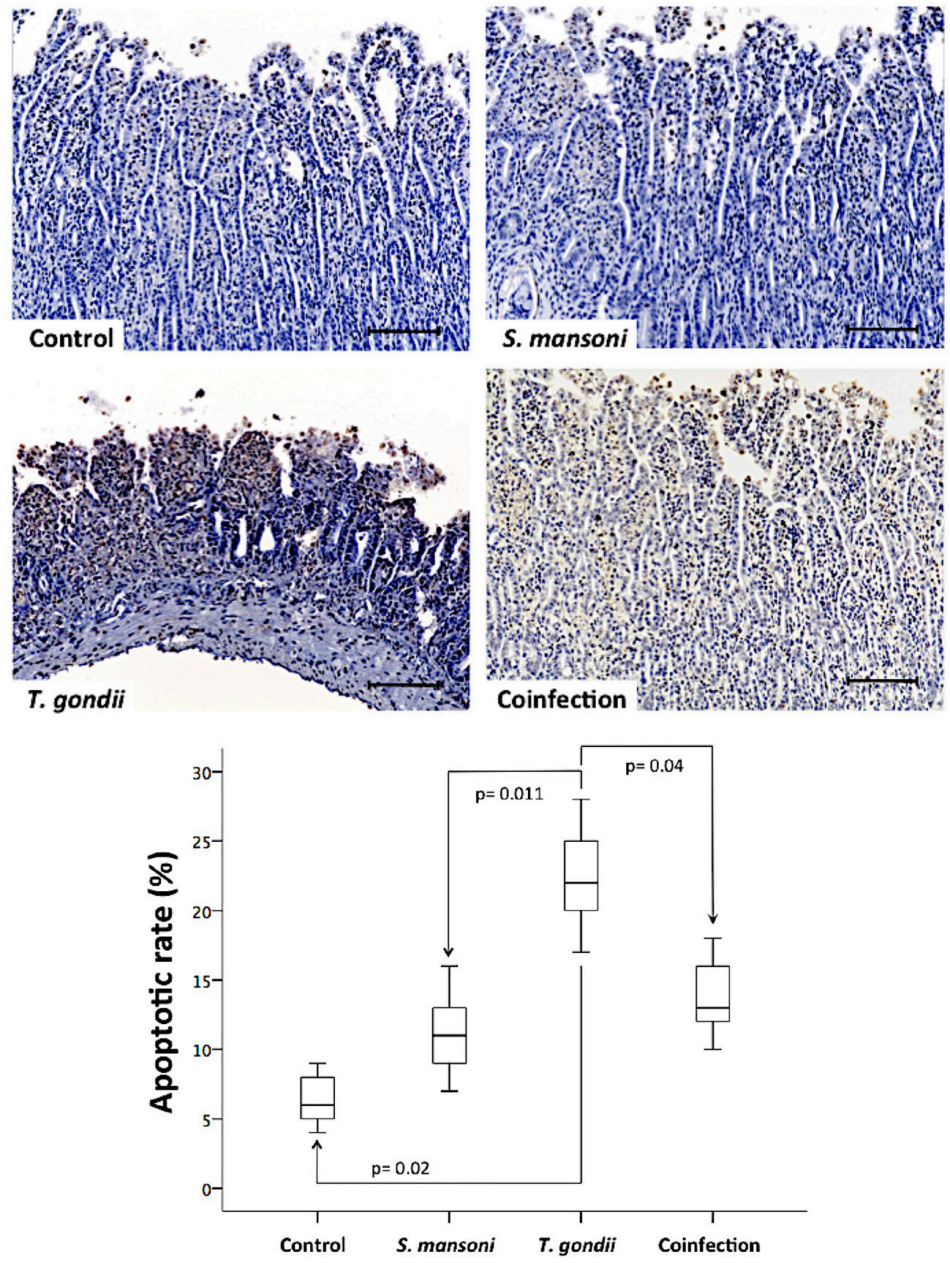
Prior *S. mansoni* infection protects the ileal mucosa against the apoptotic cell loss induced by *T. gondii*. Apoptotic cells in the terminal ileum were detected using a TUNEL assay, as shown by the representative photomicrographs. The horizontal bars represent the medians, the boxes represent the 25th and 75th percentiles, and the vertical lines below and above the boxes represent the minimum and maximum values, respectively. The analysis was performed by Kruskal-Wallis ANOVA on ranks test, in which multiple comparisons were carried out using the Dunnett's test. The scale bars represent 20 μm. The data are representative of three independent experiments, with 5–7 animals per group.

To analyze the role of cell death in tissue damage in this model, we used a TUNEL assay to label apoptotic cells. Mucosal samples from *T. gondii*-infected animals showed significantly higher rates of apoptosis compared to samples from controls and *S. mansoni*-infected animals (Figure 5).

**Figure 6:**
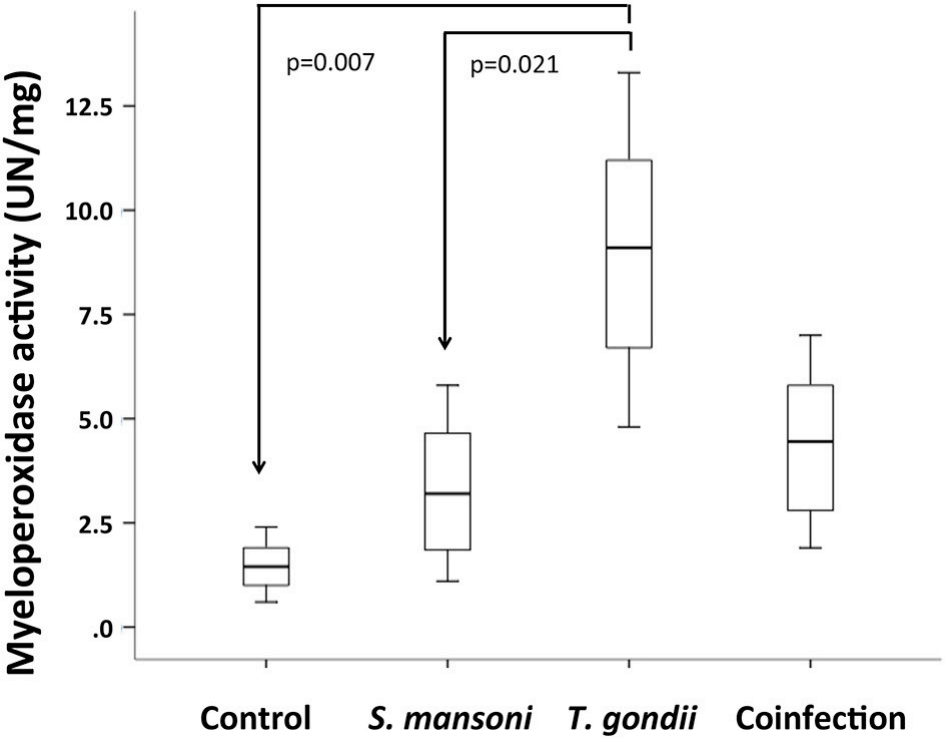
*T. gondii*-induced ileitis is characterized by an increased activity of myeloperoxidase (MPO), compared to *S. mansoni* monoinfected and control mice. Prior *S. mansoni* infection attenuates MPO activity in the terminal ileum explants of coinfected mice. The horizontal bars represent the medians, the boxes represent the 25th and 75th percentiles, and the vertical lines below and above the boxes represent the minimum and maximum values, respectively. The analysis was performed by Kruskal-Wallis ANOVA on ranks test, in which multiple comparisons were carried out using the Dunnett's test. The data are representative of two independent experiments, with 5–7 animals per group.

To analyze the participation of neutrophils in the inflammatory response in this model, we measured MPO activity in ileal explants. While MPO activity was significantly higher in *T. gondii*-infected, prior *S. mansoni* infection remarkably attenuated MPO activity in the terminal ileum explants of coinfected mice (Figure 6).

### Concurrent Infection With *S. mansoni* Controls the *T. gondii*-Mediated Inflammatory Response

**Figure 7:**
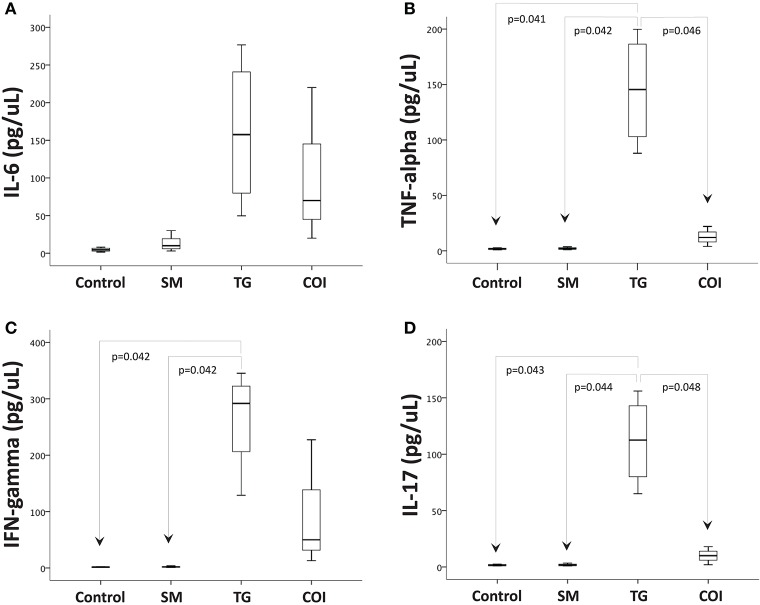
Prior *S. mansoni* infection modulates the proinflammatory cytokine production induced by *T. gondii* in the terminal ileum explants. Ileum explants were cultured for 24 h at 37°C with 5% CO_2_. The supernatants were used to measure the concentrations of cytokines by CBA. The box plots show the levels of IL-6 **(A)**, TNF-alpha **(B)**, IFN-gamma **(C)**, and IL-17 **(D)**. The horizontal bars represent the medians, the boxes represent the 25th and 75th percentiles, and the vertical lines below and above the boxes represent the minimum and maximum values, respectively. The analysis was performed by Kruskal-Wallis ANOVA on ranks test, in which multiple comparisons were carried out using the Dunnett's test. The data are representative of three independent experiments, with 5–7 animals per group.

Next, we investigated whether concurrent infection with *S. mansoni* could affect the production of inflammatory mediators mechanistically involved in the intestinal inflammation associated with *T. gondii* oral infection. For this purpose, we used a commercial mouse Th1/Th2/Th17 cytokine kit, which is based on a bead array technology to simultaneously detect several cytokine proteins in samples (IL-2, IL-4, IL-6, IFN-gamma, TNF, IL-17A, and IL-10). Here, we present only the quantifiable results obtained. The analysis of the supernatants obtained from ileal explant cultures revealed that the concentrations of IFN-gamma, TNF-alpha, and IL-17 were significantly increased in samples from *T. gondii*-infected mice compared to samples from the controls and *S. mansoni*-infected animals. The concentrations of IL-6 were not significantly different between the experimental groups, although we detected a tendency toward increased expression in the *T. gondii*-infected samples (Figure 7).

To investigate potential impacts on the systemic response, we also measured cytokine concentrations in the plasma samples obtained from the peripheral blood in a dynamic fashion. Although the results were not statistically significant, we identified a tendency toward an early elevation in IFN-gamma expression (day 4), a late elevation in TNF-alpha expression, and a possible late elevation in IL-6 expression (day 7) in the *T. gondii*-monoinfected animals compared to the controls and *S. mansoni*-infected animals (Supplementary Figure S2).

### Concurrent Infection With *S. mansoni* Modulates the Expression of Genes Involved in *T. gondii*-Induced Ileitis

**Figure 8:**
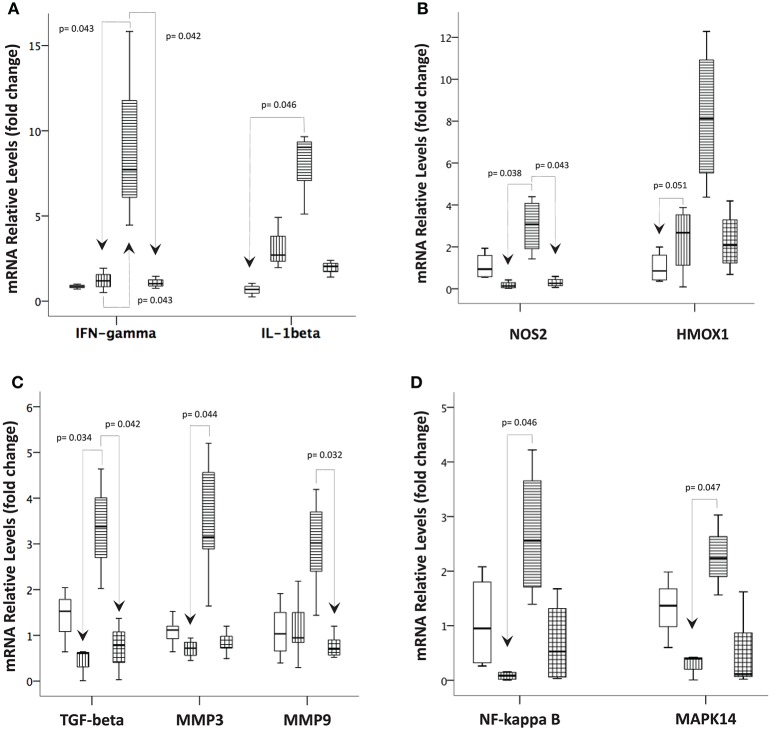
Prior *S. mansoni* infection modulates the expression of genes involved in the immune response, oxidative stress, tissue remodeling, and intracellular signaling pathways affected by *T. gondii*. Quantitative real-time PCR was used to measure the levels of **(A)** *IFN-gamma* and *IL-1 beta*; **(B)** *NOS2* and *HMOX1*; **(C)** *TGF-beta, MMP3*, and *MMP9*; and **(D)** *NF-kappa B* and *MAPK14* in the terminal ileum biopsies of the control, *T. gondii*-infected, *S. mansoni*-infected and coinfected mice. The horizontal bars represent the medians, the boxes represent the 25th and 75th percentiles, and the vertical lines below and above the boxes represent the minimum and maximum values, respectively. The analysis was performed by Kruskal-Wallis ANOVA on ranks test, in which multiple comparisons were carried out using the Dunnett's test. The data are representative of three independent experiments, with 5–7 animals per group.

To investigate the mechanism by which the infections might regulate the inflammatory response in the intestinal mucosa, we examined the mRNA expression of several genes potentially involved in the inflammatory process and tissue remodeling. Overall, the mRNA levels were increased for all target genes studied in *T. gondii*-infected ileal samples compared with samples from the other groups. For example, *IFN-gamma* mRNA expression followed the results obtained from the protein measurements in the supernatants of the explant cultures. The *IL-1 beta* and *HMOX1* mRNA levels were significantly higher in the *T. gondii*-infected samples than in the control samples. On the other hand, the *TGF-beta* and *NOS2* mRNAs displayed significantly higher levels in samples from the *T. gondii*-infected mice compared to those from *S. mansoni*-infected mice. Moreover, in samples from *T. gondii*-infected mice, the mRNA levels of *MMP3, NF-kappa B*, and *MAPK14* were higher than those in samples from *S. mansoni*-monoinfected mice, while the mRNA levels of *MMP9* were higher in samples from the *T. gondii*-monoinfected mice than samples from the coinfected mice (Figure 8).

### Concurrent Infection With *S. mansoni* Blunts *T. gondii*-Mediated NF-Kappa B and MAP Kinase Activation

**Figure 9:**
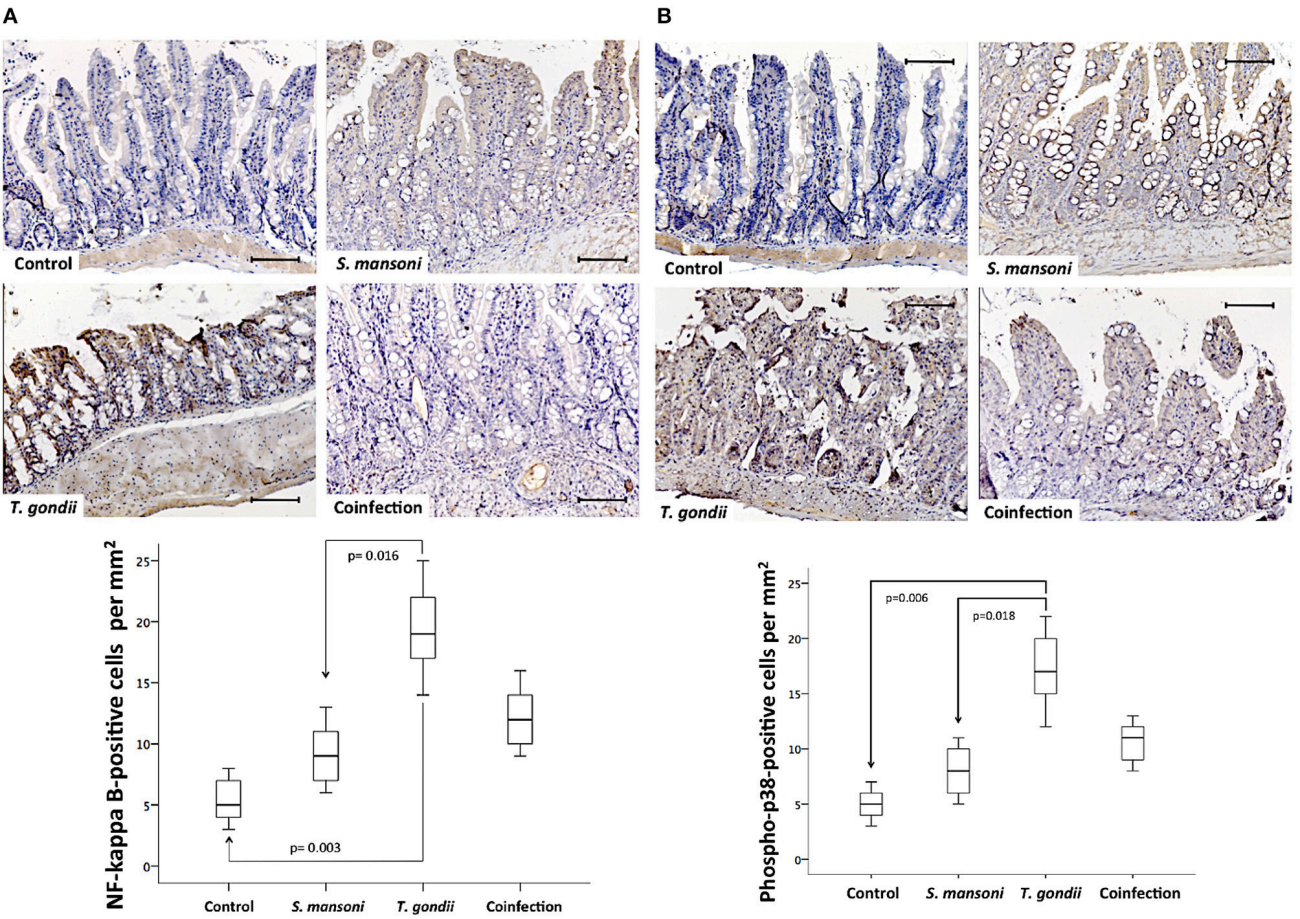
Prior *S. mansoni* infection attenuates the expression of intracellular signaling pathways involved in cytokine production and cell survival triggered by *T. gondii*. The NF-Kappa B **(A)** and phospho-p38 **(B)** expression levels are shown. The horizontal bars represent the medians, the boxes represent the 25th and 75th percentiles, and the vertical lines below and above the boxes represent the minimum and maximum values, respectively. The analysis was performed by Kruskal-Wallis ANOVA on ranks test, in which multiple comparisons were carried out using the Dunnett's test. The scale bars represent 20 μm. The data are representative of three independent experiments, with 5–7 animals per group.

To confirm the findings obtained with qPCR, the NF-kappa B and p38 MAP kinase intracellular signaling pathways were also investigated at the protein level by immunohistochemistry. NF-kappa B and phosphorylated p38 MAPK displayed similar expression patterns and tissue distributions and were present in both the epithelium and the lamina propria mononuclear cells at significantly higher densities in ileal samples from *T. gondii*-infected mice compared to those from non-infected normal controls and *S. mansoni*-infected animals (Figure 9).

## Discussion

In the present study, we investigated for the first time the possible effect of concurrent infection with *S. mansoni* on experimental *T. gondii*-induced ileitis, which is proposed as an experimental model for human CD ileitis. Oral infection with *T. gondii* induced a severe inflammatory process in the terminal ileum, with an intense inflammatory cell infiltrate, cell death, disruption of epithelial integrity with Paneth, and goblet cell depletion, and the activation of signaling pathways resulting in the production of several proinflammatory mediators and a predominant Th1/Th17-type immune response. Notably, concurrent infection with *S. mansoni* remarkably attenuated the inflammatory process and tissue destruction resulting from oral infection with *T. gondii*.

Experimental IBD is usually generated by chemical induction or immunologic or gene targeting, rendering important limitations regarding the resemblance to the human disease (17). Moreover, in the majority of these models, inflammation develops only in the colon (27, 28). On the other hand, human CD predominantly affects the terminal ileum (29, 30), and the evidence, including genome-wide association studies, supports the suggestion that CD ileitis may actually represent a distinct form of IBD (31). In this study, we worked with a model in which oral *T. gondii* infection drives an intestinal inflammatory response similar to that observed in human CD (32). Based on the previous knowledge that infections with helminths, such as *S. mansoni*, can induce a polarization of the immune response toward a Th2 phenotype with enhanced IL-4, IL-5, and IL-13 production (33) and that a soluble antigen from the *S. mansoni* egg may inhibit the production of IL-12 by dendritic cells (34), we hypothesized that concurrent infection with *S. mansoni* could be able to control or even neutralize the Th1 immune response induced by *T. gondii* infection. In fact, this study shows that *S. mansoni* coinfection appears to preserve the morphological structure of the intestine, including the epithelial layer with all the specialized cells and to control the accumulation of inflammatory cells in the lamina propria compared to *T. gondii* monoinfection.

The intestinal alterations induced by *S. mansoni* alone affected both the small bowel and the colon, resulting in granuloma formation, but with practically no effect on the lamina propria or the epithelial layer. *S. mansoni* granulomas were sparsely distributed throughout the gut, with a typical cellular infiltrate composed of a large number of eosinophils, as expected. Although the exact mechanism by which *S. mansoni* modulates *T. gondii* aggression is not fully elucidated, the enhanced presence of eosinophils, *per se*, may provide protection not only by contributing to parasite destruction but also by regulating important biological functions, such as immunoregulation (35). Interestingly, a recent study by Arnold et al. (36) further confirmed the importance of eosinophils in the regulation of Th1 responses via the IFN-gamma–dependent upregulation of PD-L1, in addition to the bactericidal properties associated with degranulation. Together, these data appear to support the role of eosinophils in the regulation of homeostatic processes within the gut and in antibacterial defense. The novel antibacterial effect of eosinophils may prove particularly relevant here, as in most experimental models of IBD, animals usually do not develop inflammation when raised under germ-free conditions, clearly implicating the intestinal microbiota in IBD pathogenesis (17, 37).

The attenuation of *T. gondii*-induced intestinal inflammation presented in this study is consistent with the protective effect of *S. mansoni* observed in a previous investigation on experimental IBD using the haptenating agent 2,4,6-trinitrobenzene sulfonic acid (TNBS), which induces a predominantly Th1-mediated response (18). Moreover, similar to human CD, the TNBS-induced colitis model results in transmural inflammation, characterized by lamina propria infiltration with CD4-positive T cells, macrophages, and neutrophils (38, 39). Regarding neutrophils accumulation and activity, similar to TNBS-induced colitis, in this study we showed that *T. gondii*-induced ileitis also triggers innate immune mechanisms, including increased myeloperoxidase activity, in addition to potential effects on the adaptive immune response. The accumulation of macrophages and CD4-positive T cells in the lamina propria of samples from *T. gondii*-induced ileitis is also compatible with the activation of innate and adaptive immune mechanisms, which are attenuated by concurrent infection with *S. mansoni*. However, although systemic abnormalities based on circulating cytokines were not significant in this study, we noted a tendency toward an increase in Th1 cytokine expression. Nonetheless, it is important to highlight that the higher rates of death in the present study occurred among the coinfected mice. The increased mortality can be explained by our results concerning liver damage, corroborating the findings of a previous study, which showed a remarkable exacerbation in liver injury, probably due to a synergistic effect of *T. gondii* and *S. mansoni* concurrent infection (40).

Although it is well established that mucosal immune tolerance depends on the equilibrium between effector and regulatory T cells (41), results from various investigations, particularly genome-wide association studies performed in the last decade, have reinforced the importance of innate immune and epithelial cells in the pathogenesis of CD (42). In addition to composing a physical barrier, the intestinal epithelium also possesses specialized cells, such as goblet cells and Paneth cells, which are able to secrete protective mucous and antimicrobial peptides, respectively (43). Goblet cells secrete mucins that form the gel constituting the intestinal mucous, which comprises a dense and sterile inner layer and a more permeable external layer populated by commensal microorganisms (44). The relationship between the microbiota and the intestinal mucus is still incompletely understood, but there is evidence of a reciprocal interaction, as mucus permeability was shown to depend on the composition of the microbiota, and vice versa (45).

A distinctive feature of the small bowel is the remarkable presence of Paneth cells within the epithelium, lying in the bottom of the Lieberkühn crypts, which have a characteristic antimicrobial function involving the secretion of peptides, such as alpha-defensins (46). The role of Paneth cells in the control of the microbiota has been reinforced by several experimental studies in which cell depletion resulted in microbial accumulation in the lamina propria and mesenteric lymph nodes (47) and susceptibility to infections by the oral route (48). The evidence linking Paneth cells to CD is rather complex and comprises several genetic, microbial and functional aspects. The first hint suggesting a role for Paneth cells in CD came from the observation that *NOD2*, the first gene polymorphism associated with CD, is highly expressed in Paneth cells (49). Another study described a reduction in the synthesis of defensins by Paneth cells in patients with ileal CD (50). In a British study, the reduction in the production of defensins in patients with CD was shown to be more relevant in a cohort bearing a *NOD2* mutation (51). Possible defects in Paneth cell maturation and differentiation may also be important in the pathogenesis of CD. For example, in patients with ileal CD, low expression of Wnt and TCF-4 has been detected, regardless of the presence of inflammation (52). Moreover, the abnormal granule exocytosis observed in *ATG16L*-deficient animals (53), further supports a role for defects in Paneth cells in CD, as an *ATGL16* gene mutation has also been associated with CD (54).

Regarding the mechanisms underlying the inflammatory process and the immune response, the results of this study appear to support the notion that *T. gondii*-induced ileitis drives a predominant Th1 and Th17 response, with high concentrations of IFN-gamma, TNF-alpha, IL-17, and even IL-1 beta in the explants from the ileal samples. In fact, previous studies have shown that some experimental models of IBD are based on both Th1 and Th17 responses (17, 55), similar to what is observed in human CD (56). Here, IL-6 did not differ significantly among groups and, transforming growth factor (TGF)-beta, the only with direct and consistent anti-inflammatory potential among the molecules analyzed, displayed a similar behavior as TNF-alpha, IL-17, IFN-gamma, and IL1-beta. Although data on IL-2, IL-4, and IL-10 could have helped in further characterizing the immune response, the study protocol and/or the kit performance imposed limitations for the analysis of relevant concentrations of specific cytokine proteins in tissue culture supernatants and serum samples used in this study. On the other hand, the increase in IL-1 beta expression detected in this study may represent additional evidence for innate immune activation in the model in association with defects in the epithelial barrier. IL-1 beta is implicated in immediate and early innate immune responses against microbial constituents (57) and is also directly involved in the activation of the inflammasome (58). In addition, IL-1 beta has been associated with the pathogenesis of IBD, especially CD (39), and in experimental IBD, IL-1 beta has been shown to induce an increase in intestinal permeability in association with NF-kappa B activation (59).

In this model, *T. gondii*-induced ileitis also induced the activation of intracellular signaling pathways, including the NF-kappa B and p38 mitogen-activated protein kinase (MAPK) pathways, suggesting that these pathways might be involved in the underlying inflammatory process. Nonetheless, it is interesting to note that in this study, *S. mansoni* infection apparently directs an opposing response, tending to decrease the expression of both NF-kappa B and MAPK induced by *T. gondii* infection. Notably, abnormalities in intracellular signaling pathways can lead to dysregulation in the immune response favoring the perpetuation of inflammation observed in IBD in a bidirectional fashion, as proinflammatory cytokines can activate NF-kappa B (60), and NF-kappa B can orchestrate inflammation both in experimental models (61) and in human IBD (62). In addition to NF-kappa B, p38 MAPK has also been implicated in the chronic inflammatory process underlying IBD, as the pathway is capable of regulating the expression of proinflammatory cytokines, including TNF-alpha, within the intestinal mucosa of patients with CD (63) and is also involved in the local inflammatory response in experimental IBD (64). In the present study, the downregulation of NF-kappa B and phospho-p38 MAPK expression corroborates the attenuation of the inflammatory process of *T. gondii*-induced ileitis but does not clarify whether the effect of the concurrent infection with *S. mansoni* on intracellular signaling pathways is direct or indirect.

Several characteristics of the inflammatory process underlying IBD are consistent with the physiological properties of nitric oxide (NO), which has been implicated in the regulation of motility, mucosal blood flow, and epithelial protection, among other effects (65). In IBD, NO has been shown to mediate macrophage-induced senescence and the DNA damage response, particularly in CD, which presents with higher levels of NO synthase 2 (NOS2) compared to UC (66). Similar to what has been shown in human CD, *T. gondii*-infected samples also presented higher expression of *NOS2* in this study. Another potentially protective molecule analyzed in this investigation was heme oxygenase-1 (HMOX1), an enzyme with anti-inflammatory and immune regulatory functions capable of modulating immune-mediated diseases, experimental colitis (67), and human IBD (68). Together, these findings appear to reinforce the roles of NO and HMOX1 as important regulators in chronic and acute inflammatory processes and suggest their participation in the homeostatic pathways involved in IBD.

Matrix metalloproteinases (MMPs) have been linked to extracellular matrix turnover in physiological and pathological conditions. The activity of MMPs relative to that of tissue inhibitors of MMPs has been shown to be increased in inflamed samples of patients with IBD (69) and in experimental colitis (70). Interestingly, the increased spatial expression of MMPs in segments of resected terminal ileum from patients with CD has been associated with the higher recurrence of intestinal strictures (71). Here, we also identified increased expression of *MMP3* and *MMP9* in the samples from *T. gondii*-infected animals compared with those from *S. mansoni*-monoinfected or -coinfected animals. Another important molecule in tissue remodeling, TGF-beta, a pleiotropic cytokine possessing important tolerogenic action in the gut (72), also showed higher expression in *T. gondii*-induced ileitis in the current study. In fact, the intestinal mucosa from patients with CD has been shown to overexpress TGF-beta (73), particularly in areas of intestinal stricture (74), whereas *in vitro* studies demonstrated that TGF-beta promotes the production of collagen by human intestinal smooth muscle cells and myofibroblasts (75). In addition, the increased expression of TGF-beta in the mucosa overlying CD strictures was also been shown to stimulate the local production of MMPs (76). The results obtained from the ileal samples of *T. gondii*-infected animals in the present study appear to be consistent with the expected changes in the expression of MMPs and TGF-beta in acute and chronic inflammation and the abnormal tissue remodeling observed in IBD.

Collectively, our data suggest that concurrent infection with *S. mansoni* attenuates the course of *T. gondii*-induced enteritis by downregulating both the adaptive and innate immune responses and by inhibiting the NF-kappa B and p38 MAPK intracellular signaling pathways and oxidative stress-mediated mechanisms. Moreover, the preservation of Paneth cells and goblet cells in coinfected mice further supports the idea that the protection against *T. gondii*-induced ileitis could be related to mechanisms of innate immunity, with potential effects on the epithelial barrier function and the intestinal microbiota. However, the mechanistic insights to explain the observed results are premature at this stage and constitute part of our ongoing investigations. Nonetheless, the findings presented here appear to be in accordance with the evolutionary concept in which mammalian coevolution with helminths and other species have shaped immune function so that effective responses are dependent on a normal biome (77). Such a normal biome, in turn, most likely encompasses helminth colonization, with a range of direct and indirect influences resulting in the downregulation of the immune system (78). Therefore, we speculate that helminth colonization enhances the production of regulatory elements and diminishes the propensity for immune-mediated disorders. In the context of chronic inflammatory diseases with predominant Th1/Th17 responses, *S. mansoni* or its constituents emerge as a conceptually novel approach to treatment, potentially including the treatment of CD.

## Data Availability

The datasets generated for this study are available on request to the corresponding author.

## Author Contributions

BP and CM participated in the conception and design of the study; the acquisition, analysis, and interpretation of data; and the drafting of the manuscript. CB, AdAC, JdSM, HN, BR, and MM participated in the acquisition, analysis, and interpretation of the data and the drafting of parts of the manuscript. MC-B participated in the design of the study and the analysis and interpretation of the data and critically revised the manuscript for important intellectual content. MC and HdS participated in the conception and design of the study; obtained funding; analyzed and interpreted the data; and critically revised the manuscript for important intellectual content. All authors gave final approval of the submitted version of the manuscript.

### Conflict of Interest Statement

The authors declare that the research was conducted in the absence of any commercial or financial relationships that could be construed as a potential conflict of interest.

## Supplementary Material

Supplementary Figure S1 *S. mansoni* monoinfection resulted in large multifocal eosinophilic granulomas in the liver, although the essentially normal structure was preserved. Liver sections from *T. gondii*-infected mice displayed foci of inflammation, with a relative preservation of the general structure. In coinfected mice, larger and less eosinophilic granulomas, surrounded by extensive areas of coagulative necrosis and hepatocyte vacuolization were observed. The scale bars represent 10 μm. **(A)** Significantly increased AST and ALT, liver-associated enzymes, were detected in the serum of coinfected mice, compared to the other experimental groups. Levels in coinfected animals were almost twice as much of those observed among *T. gondii*-infected mice (at day 7) **(B)**. The horizontal bars represent the medians, the boxes represent the 25th and 75th percentiles, and the vertical lines below and above the boxes represent the minimum and maximum values, respectively. The analysis was performed by Kruskal-Wallis ANOVA on ranks test, in which multiple comparisons were carried out using the Dunnett's test. The data are representative of two independent experiments, with 3–5 animals per group.

Supplementary Figure S2 Expression levels of early systemic pro- and anti-inflammatory cytokines are upregulated during coinfection. Plasma samples were used to measure the concentrations of cytokines by CBA: IL-10 **(A)**, IL-17A **(B)**, TNF-α **(C)**, IFN-γ **(D)**, IL-6 **(E)**, IL-4 **(F)**, and IL-2 **(G)**. The analysis was performed by linear regression. Red arrows indicate the highest systemic cytokine levels at day 4. Black arrows point to the highest level of cytokine secretion on day 7. DPI, days postinfection; CTL, control; Sm, *S. mansoni* infection; Tg, *T. gondii* infection; COI, coinfection with *S. mansoni* and *T. gondii*. The data are representative of two independent experiments, with 3–5 animals per group.
