# Supplementary figures and images for: Schistosoma mansoni Coinfection Attenuates Murine Toxoplasma gondii-Induced Crohn's-Like Ileitis by Preserving the Epithelial Barrier and Downregulating the Inflammatory Response
Source: Front Immunol. 2019 Mar 18;10:442. doi: 10.3389/fimmu.2019.00442 (PMC6432985; doi:10.3389/fimmu.2019.00442)

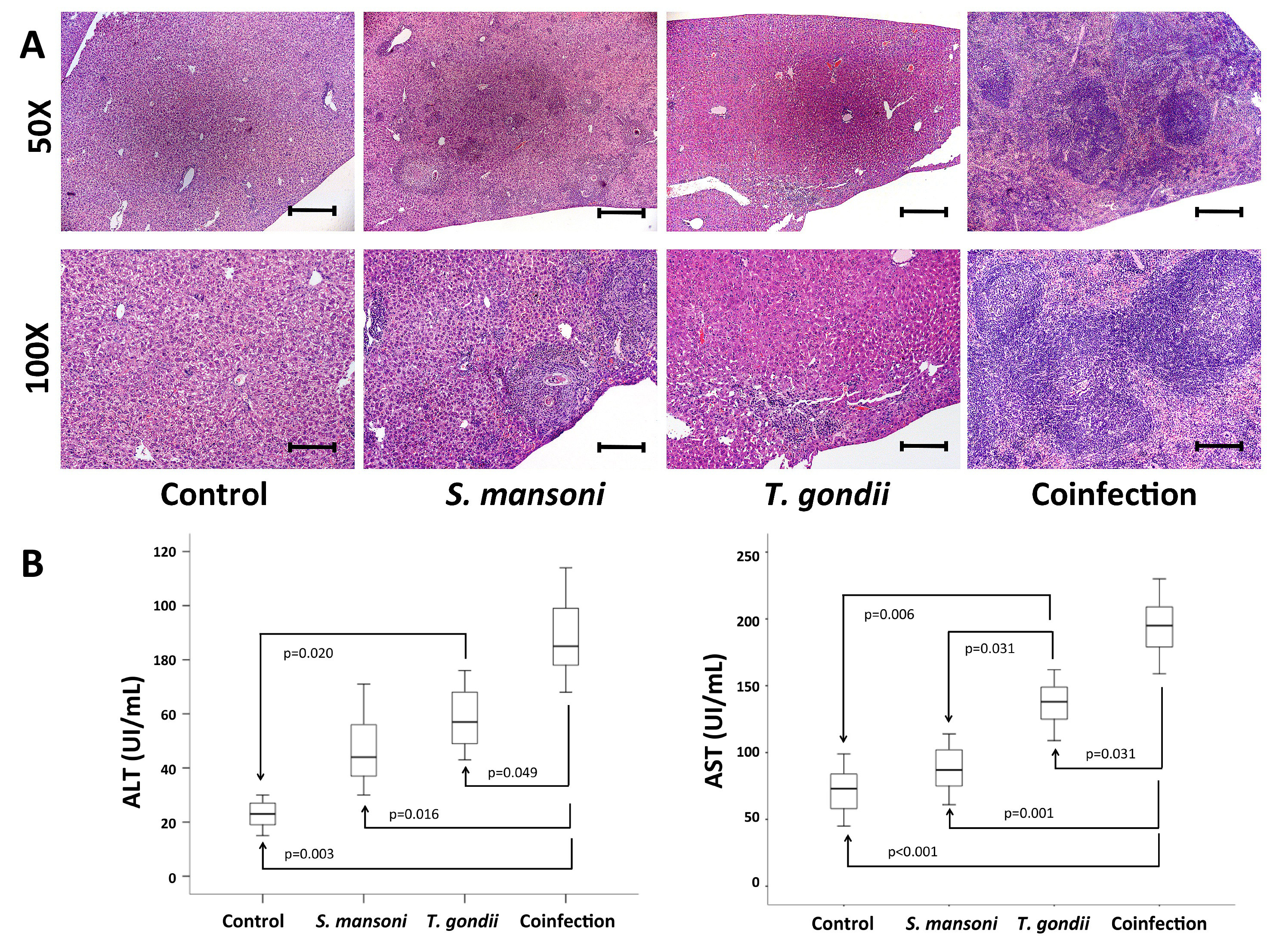

Supplement: Supplementary Figure S1 — S. mansoni monoinfection resulted in large multifocal eosinophilic granulomas in the liver, although the essentially normal structure was preserved. Liver sections from T. gondii-infected mice displayed foci of inflammation, with a relative preservation of the general structure. In coinfected mice, larger and less eosinophilic granulomas, surrounded by extensive areas of coagulative necrosis and hepatocyte vacuolization were observed. The scale bars represent 10 μm. (A) Significantly increased AST and ALT, liver-associated enzymes, were detected in the serum of coinfected mice, compared to the other experimental groups. Levels in coinfected animals were almost twice as much of those observed among T. gondii-infected mice (at day 7) (B). The horizontal bars represent the medians, the boxes represent the 25th and 75th percentiles, and the vertical lines below and above the boxes represent the minimum and maximum values, respectively. The analysis was performed by Kruskal-Wallis ANOVA on ranks test, in which multiple comparisons were carried out using the Dunnett's test. The data are representative of two independent experiments, with 3–5 animals per group. [file Image_1.TIF]

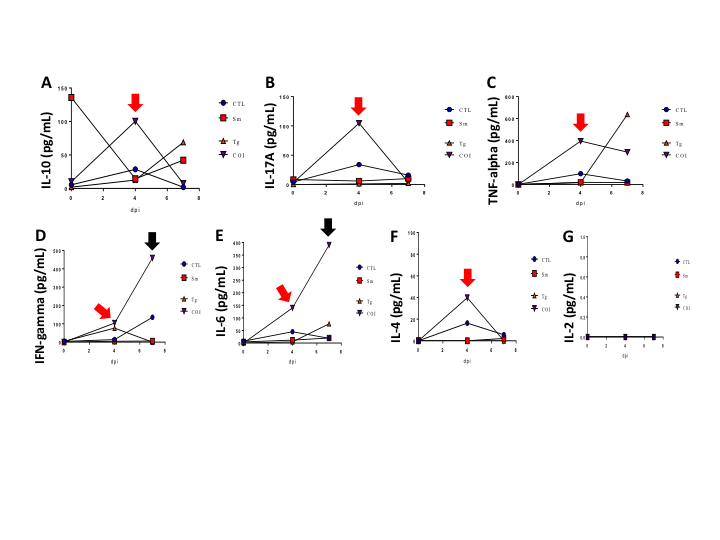

Supplement: Supplementary Figure S2 — Expression levels of early systemic pro- and anti-inflammatory cytokines are upregulated during coinfection. Plasma samples were used to measure the concentrations of cytokines by CBA: IL-10 (A), IL-17A (B), TNF-α (C), IFN-γ (D), IL-6 (E), IL-4 (F), and IL-2 (G). The analysis was performed by linear regression. Red arrows indicate the highest systemic cytokine levels at day 4. Black arrows point to the highest level of cytokine secretion on day 7. DPI, days postinfection; CTL, control; Sm, S. mansoni infection; Tg, T. gondii infection; COI, coinfection with S. mansoni and T. gondii. The data are representative of two independent experiments, with 3–5 animals per group. [file Image_2.TIF]
